# Kinesin-5 Contributes to Spindle-length Scaling in the Evolution of Cancer toward Metastasis

**DOI:** 10.1038/srep35767

**Published:** 2016-10-21

**Authors:** Ching-Feng Yang, Wan-Yu Tsai, Wei-An Chen, Kai-Wen Liang, Cheng-Ju Pan, Pei-Lun Lai, Pan-Chyr Yang, Hsiao-Chun Huang

**Affiliations:** 1 Institute of Molecular and Cellular Biology, National Taiwan University, Taipei 10617, Taiwan; 2 Department of Electrical Engineering, National Taiwan University, Taipei 10617, Taiwan; 3 Genome and Systems Biology Degree Program, National Taiwan University, Taipei 10617, Taiwan; 4 Department of Internal Medicine, National Taiwan University, Taipei 10617, Taiwan; 5 Department of Life Science, National Taiwan University, Taipei 10617, Taiwan; 6 Graduate Institute of Electronics Engineering, National Taiwan University, Taipei 10617, Taiwan

## Abstract

During natural evolution, the spindles often scale with cell sizes to orchestrate accurate chromosome segregation. Whether in cancer evolution, when the constraints on genome integrity are relaxed, cancer cells may evolve the spindle to confer other advantages has not been investigated. Using invasion as a selective pressure *in vitro*, we found that a highly metastatic cancer clone displays a lengthened metaphase spindle, with faster spindle elongation that correlates with transiently elevated speed of cell migration. We found that kinesin-5 is upregulated in this malignant clone, and weak inhibition of kinesin-5 activity could revert the spindle to a smaller aspect ratio, decrease the speed of spindle pole separation, and suppress post-mitotic cell migration. A correlation was found between high aspect ratio and strong metastatic potential in cancers that evolved and were selected *in vivo*, implicating that the spindle aspect ratio could serve as a promising cellular biomarker for metastatic cancer clones.

Whether (and, how) spindles scale with cell sizes has been a central question in biology, as this phenomenon is crucial for the quantitative understanding of cell division. The metaphase spindle is a dynamic steady-state structure composed of microtubules and hundreds of accessory proteins. For a spindle to efficiently search and accurately capture the kinetochores in a mitotic cell to divide the genome, it should, in theory, scale with cell size. Indeed, current consensus is that spindle length increases with the dimension of the dividing cell during ontogeny and phylogeny^1^,^2^,^3^,^4^,^5^,^6^,^7^,^8^. Imaging of spindles in intact cells across evolutionarily closed species (e.g., nematodes^3^,^6^) and metazoans^5^ has been the mainstay method for the study of spindle scaling. *Xenopus* egg extract is another attractive model system to investigate how, in natural evolution, spindles may scale with cell sizes^1^,^2^. In this system, inhibitory phosphorylation of Katanin was identified to be responsible for spindle scaling in *X. laevis* (larger frog) and *X. tropicalis* (smaller frog)^2^.

Cancer is thought to be a disease of clonal evolution in the human body^9^,^10^,^11^. The constraints on genome integrity are relaxed in a dividing somatic cancer cell to promote neoplastic progression, resulting in a large genetically or epigenetically heterogeneous population of cells^9^,^11^. Clonal evolution of cancer generally selects cells with increased proliferation and better survival, invasion, and metastasis^9^,^11^. With repeated rounds of *in vitro* selection of subclones originating from the same primary lung adenocarcinoma (Fig. 1A), we have previously established a panel of phenotypically stable lung cancer cell lines (CL) with differential metastatic potential^12^. In short, CL1 was initially established from a single-cell clone and became heterogeneous, presumably due to the genomic instability characteristic of cancer. Evolved, metastatic subpopulations from CL1 were collected and expanded into six lines with progressive metastatic potency, designated as CL1-0 (parental, the least metastatic), CL1-1, CL1-2, CL1-3, CL1-4, and CL1-5 (the most metastatic) (Fig. 1A). This panel of model cell lines has enabled the genome-wide identification of multiple differentially expressed genes that were later confirmed to associate with cancer metastasis^13^,^14^,^15^,^16^,^17^. For instance, with the CL series, collapsin response mediator protein-1 (CRMP-1) was identified as a novel metastasis-suppressing gene^14^. CRMP-1 is highly expressed in the least metastatic CL1-0 to depolymerize F-actin, inhibit filopodia formation, and thereby, suppress cell migration^14^,^18^.

We reasoned that the CL series might present an alternative model system for the study of spindle scaling in the case of cancer evolution toward metastasis. Conceptually parallel to experimental evolution^10^, here, invasion was used as the selective pressure *in vitro* to isolate a series of subclones to serve as genetic variants. With this model, we investigated whether the spindle-scaling rule is still preserved in this context, and if not, whether cancer cells may evolve the spindle to confer other advantages in metastasis. We focused on two extreme clones within the CL series, CL1-0 and CL1-5. We found that the metastatic CL1-5 accommodated a lengthened metaphase spindle due to an upregulation of kinesin-5, a motor protein that pushes interpolar microtubules apart. Dynamically, this kinesin-5 upregulation led to faster spindle elongation in anaphase B, which correlated with a transiently elevated speed and directional persistence of post-mitotic cell migration. This, and the fact that faithful DNA segregation is no longer a priority for dividing cancer cells, may allow the maintenance of lengthened or even deformed spindles in highly metastatic cancer clones.

## Results

### Metastatic CL1-5 cells accommodate relatively lengthened spindles

**Figure 1:**
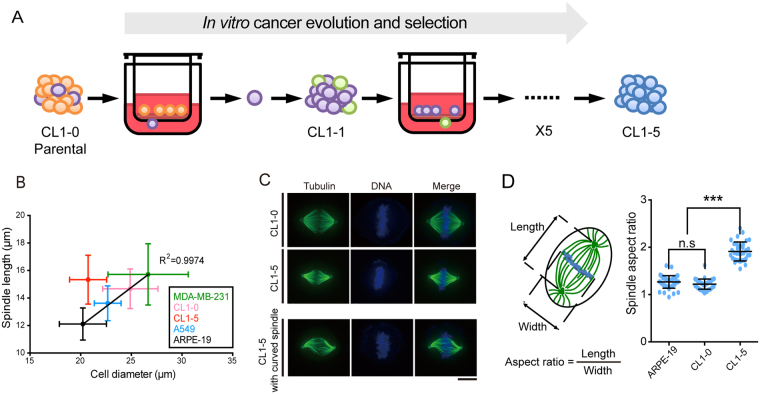
Evolution and Selection of Lengthened Mitotic Spindles in CL Series. (**A**) Schematic diagram of *in vitro* cancer evolution and selection of CL series. Evolved, metastatic subpopulations of CL1 were collected and expanded by repeated rounds of *in vitro* selection using Transwell invasion chambers. CL1-0: parental line. CL1-5: the most invasive line after five rounds of expansion and selection. (**B**) Average metaphase spindle length plotted against average cell diameter for the five human cell lines CL1-0, CL1-5, A549, MDA-MB-231 and ARPE-19. Cells were synchronized by double thymidine block, released for 8 hr, and treated with MG132 for 1.5 hr to shortly arrest them at metaphase. The cells were fixed and stained for tubulin and DNA, and only cells with aligned chromosomes were considered at metaphase. Data are represented as the mean ± SD (n = 46, 34, 28, 26 and 64 for CL1-0, CL1-5, A549, MDA-MB-231 and ARPE-19, respectively). (**C**) Representative immunofluorescence images of CL1-0 (top), CL1-5 (middle), and curved CL1-5 spindles (bottom). Tubulin: green; DNA: blue. Scale bar: 10 μm. (**D**) Spindle aspect ratio of ARPE-19, CL1-0 and CL1-5. Aspect ratio is defined as spindle length (pole-to-pole distance) divided by width (at metaphase plate)^44^. Average aspect ratio is 1.26 ± 0.11 (n = 45), 1.22 ± 0.11 (n = 46) and 1.91 ± 0.20 (n = 34) for ARPE-19, CL1-0 and CL1-5, respectively. *P* < 0.001; Student’s *t* test.

To clarify whether the spindle architecture may be altered during the evolution of cancer metastasis, we employed the CL series of lung cancer cell lines with progressive metastatic capacity^12^,^13^,^14^,^15^,^16^,^17^ (Fig. 1A). We chose to focus on CL1-0 and CL1-5, the least and the most metastatic clone, respectively, within the series (Fig. 1A). CL1-5 exhibits significantly higher *in vivo* tumorigenicity and metastatic potential than CL1-0^12^. We confirmed that CL1-5 had much higher migration potential than CL1-0 by using a Transwell migration assay (Figure S1A) and by live-cell imaging to track individual cell migration (Figure S1B; CL1-0 cells were generally immobile, whereas CL1-5 cells tended to migrate over long distances). To first assess whether the spindles scale with cell sizes in the two CL lines, we defined cell size as the cell diameter during mitosis, and spindle length was measured at metaphase. CL1-5 cells were slightly smaller than CL1-0 cells as reported previously^12^ (Fig. 1B). However, the CL1-5 metaphase spindle was longer than that in CL1-0 (Fig. 1B). To position CL1-0 and CL1-5 on the size-length relationship of human cells, we included two commonly used cancer cell lines, A549 (lung cancer) and MDA-MB-231 (breast cancer), as well as a normal cell line ARPE-19 (human retinal pigment epithelial cell). We found that the average spindle length correlated very well with the average cell size for A549, CL1-0, MDA-MB-231, and ARPE-19 (Fig. 1B, R^2^ = 0.9974; notably, there was significant intra-line variation, and spindle length correlated less well with cell size among individual cells, Figure S2). CL1-5, on average, evolved a rather lengthened spindle given its relatively small size (Fig. 1B). The spindles of both lines were morphologically distinct (Fig. 1C; Videos S1 and S2 for 3D reconstructed spindles). The aspect ratio, defined as the spindle length divided by the width at metaphase, was significantly higher in CL1-5 than in CL1-0 (Fig. 1D, note that there was no significant difference between CL1-0 and ARPE-19), and slightly increased in CL1-1 and CL1-2 (Figure S3). In 39% of cases (n = 142), CL1-5 spindles even curved at the cell periphery (Fig. 1C; Video S3 for 3D reconstructed spindles), suggesting that the spindle poles may be pushed apart in CL1-5^19^. In CL1-5 cells, attachment of the chromosomes to the spindles tended to take longer time after the bipolar spindle had been established (longer duration of mitosis, Figure S4A; Video S4 and S5 for representative CL1-0 and CL1-5 cells at prometaphase), and the cells displayed an increased frequency of lagging chromosomes during anaphase (Figure S4B), possibly due to the deformation of the spindles.

### CL1-5 cells exhibit increased spindle elongation rates and migration speeds

**Figure 2:**
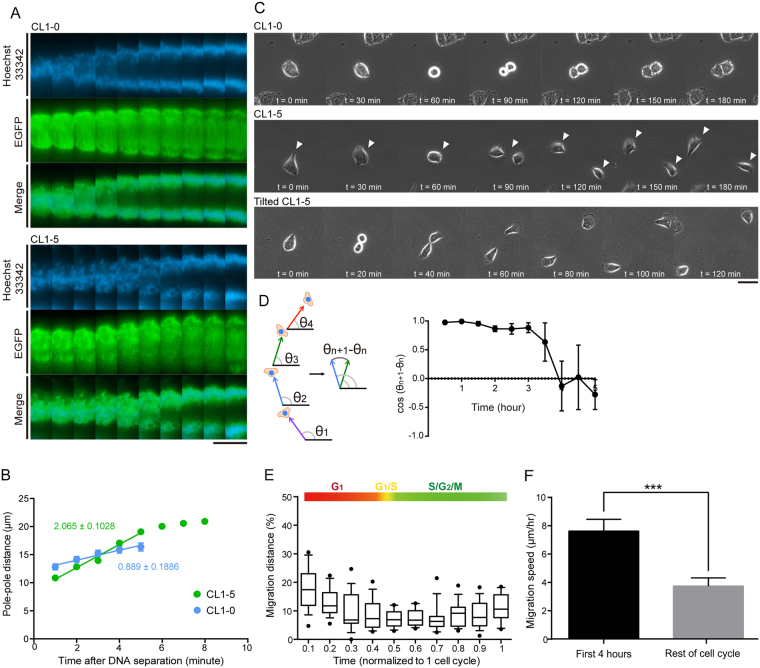
Spindle Dynamics Promotes Migration in CL1-5. (**A**) Representative kymograph of CL1-0 (top) and CL1-5 (bottom) spindles and chromosomes labeled with GFP-tubulin (green) and Hoechst 33342 (blue), respectively. Fluorescence images were taken every minute. Scale bar: 30 μm. (**B**) Pole-pole distance as a function of time for CL1-0 (blue) and CL1-5 (green) starting from metaphase until spindle poles were decomposed. Data are represented as the mean ± SEM (n = 15 and 10 for CL1-0 and CL1-5, respectively). (**C**) Representative time-lapse images of CL1-0 (top), CL1-5 (middle), and tilted CL1-5 (bottom) migration following cytokinesis. Phase-contrast images were taken every 30 (top and middle) or 20 (bottom) min. Scale bar: 50 μm. (**D**) Transient directional persistence of CL1-5 migration following mitosis. Schematic diagram (left) illustrates a hypothetical cell trajectory. *θ*_*n+1*_ − *θ*_*n*_ is the angle between two sequential time steps, and cos (*θ*_*n+1*_ − *θ*_*n*_) indicates whether a cell persists to migrate in its previous direction (cos (*θ*_*n+1*_ − *θ*_*n*_) = 1: same direction). Data are represented as the mean ± SEM (at early time points, SEMs are too small to be visible; n = 17 cells). (**E**) Boxplot of the speed of CL1-5 single-cell migration profiled over the course of cell cycle. The percentage of migrated distance (distance migrated in the indicated time interval divided by total distance migrated) was plotted against time in the cell cycle (each time step represents one tenth of the cell cycle). Migration of individual CL1-5 cells was monitored from mitosis (after cytokinesis, t = 0) to mitosis (before mitotic entry, t = 1). n = 16 cells. (**F**) Comparison of migration speed between the post-mitotic four hr and the rest of the cell cycle in individual CL1-5 cells. Data are represented as the mean ± SEM.

We reasoned that this aberrant spindle architecture may have conferred functional advantage with respect to metastasis for it to be selected in CL1-5 (a neutral “hitchhiker”^9^). We investigated the possible correlation between spindle dynamics and cell migration. To this end, we measured the pole-to-pole distance as a function of time in stable GFP-tubulin-expressing CL1-0 and CL1-5, starting from metaphase (judged by chromosome alignment at the metaphase plate) until the spindle poles were no longer visible. The speed of spindle elongation was significantly higher in CL1-5 than in CL1-0 (Fig. 2A,B, linear regression slopes are 2.065 ± 0.1028 and 0.8888 ± 0.1886 for CL1-5 and CL1-0, respectively). Following cell division/cytokinesis, CL1-5 cells tended to jump apart, whereas CL1-0 cells flattened out and remained clustered (Fig. 2C). In some cases, CL1-5 cells migrated in a direction that was slightly tilted and not perpendicular to the plane of cleavage furrow, a phenotype that reflects the curving morphology of the spindles (Fig. 2C; Figure S5 for tubulin merged with cell morphology from GFP and phase channel, respectively). To further inspect whether a fast spindle dynamics in mitosis may correlate with cell migration in CL1-5, we analyzed trajectories of single cells (Fig. 2D). In the metastatic CL1-5 clone, due to genome instability there were 30.31% ± 4.17% of cells that were immobile and appeared CL1-0-like (quantification of CL1-5 heterogeneity for three independent passages were included in Figure S6); these cells were excluded from analysis. Following mitosis, CL1-5 cells transiently maintained their direction of migration; however, after 4 hr, the directionality was lost and the cells began to migrate randomly (Fig. 2D). Using the previously characterized CL1-5-FUCCI line^20^, we profiled cell migration over the course of the cell cycle (Fig. 2E). The percentage of migration distance of CL1-5-FUCCI cells peaked immediately after division (Fig. 2E), and the average speed over the first 4 hr (first half of G1, before cells started to lose their directional persistence) was significantly higher than during the rest of the cell cycle (Fig. 2F). The post-mitotic elevated speed and directional persistence were absent in CL1-0 (Figure S7A,B). Altogether, these data suggest that CL1-5 accommodates fast spindle elongation during anaphase B, correlating with elevated speed and transient directional persistence of cell migration.

### The expression level of kinesin-5 governs spindle scaling in the CL series

**Figure 3:**
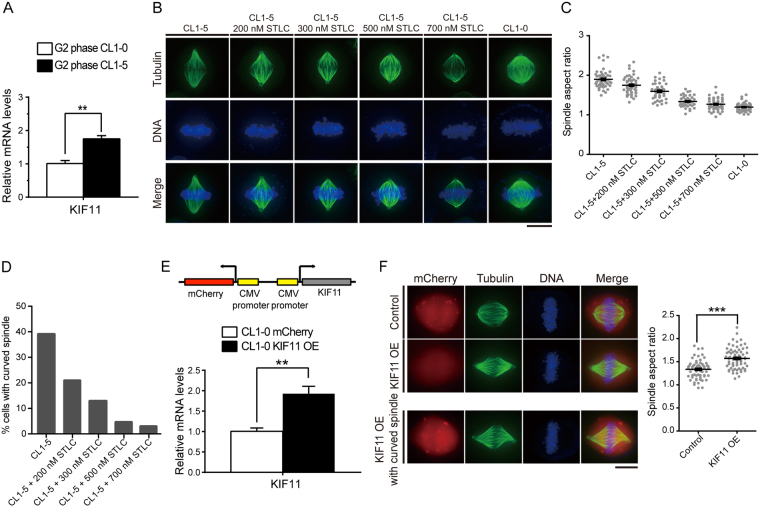
Kinesin-5 Governs Spindle Scaling in CL Lines. (**A**) Relative kinesin-5 mRNA levels in G2 phase CL1-0 and CL1-5 cells measured by qRT-PCR (*n* = 3 independent experiments, *P* < 0.01; Student’s *t* test). GAPDH was used as the reference gene. (**B**) Representative immunofluorescence images of CL1-0 and CL1-5 spindles with indicated treatments. Tubulin: green; DNA: blue. Scale bar: 10 μm. (**C**) Spindle aspect ratio of CL1-0 and CL1-5 with indicated treatments. n = 48 for each group. (**D**) Percentages of curved spindles for CL1-5 with indicated treatments. n = 142, 155, 189, 186, and 181 for CL1-5, CL1-5 treated with 200, 300, 500, and 700 nM STLC, respectively. (**E**) (Top) schematic diagram of kinesin-5 over-expression construct. The expression of untagged kinesin-5 is driven by one CMV promoter, whereas the other promoter drives the expression of mCherry to serve as the internal control for transfection. (Bottom) escalated level of kinesin-5 mRNA confirmed by qRT-PCR (*n* = 3 independently experiments, *P* < 0.001; Student’s *t* test). (**F**) (Left) representative immunofluorescence images of CL1-0 spindles with indicated treatments. Tubulin: green; DNA: blue; mCherry: red. Scale bar: 10 μm. (Right) spindle aspect ratio of CL1-0 with indicated treatments. Only cells with red fluorescent signals were scored. n = 62 and 71 for CL1-0 and CL1-0 overexpressing kinesin-5, respectively.

Cytoplasmic factors can be fine-tuned during evolution to regulate the steady-state spindle architecture^2^,^21^,^22^. For instance, in *Xenopus*, inhibitory phosphorylation of the microtubule-severing protein katanin is believed to govern interspecies spindle-length scaling^2^. We reasoned that massive cancer genome evolution might result in differences in the expression of some microtubule regulators^13^, which might lead to spindle scaling in the CL series. To screen for differentially expressed genes, we performed microarray analysis on CL1-0 and CL1-5 cells synchronized at G2 (because the expression of several microtubule regulators is cell cycle-dependent and peaks at G2/M)^23^,^24^. Kinesin-5 (KIF11, or Eg5) was one of the spindle regulators the most highly upregulated in CL1-5 (fold change = 2.73, Table S1), and the near two-fold upregulation was confirmed with quantitative reverse transcription PCR (qRT-PCR) (Fig. 3A). Kinesin-5 is a plus-end-directed, homotetrameric motor protein that cross-links microtubules^25^,^26^,^27^. In the mitotic spindle, it is believed to push apart anti-parallel interpolar microtubules and thus to be essential to mitotic spindle bipolarity^25^,^26^,^27^. Indeed, overexpression or reduction of kinesin-5 led to metaphase spindle lengthening or shortening, respectively, in yeast and *Xenopus* egg extracts^4^,^28^,^29^. However, in fly and cultured human cells, when titrating the level of kinesin-5, the mitotic spindles are either monopolar, or bipolar of fixed length^30^,^31^. In other words, above a certain threshold, the metaphase spindle length is robust to small variations in the level of kinesin-5, and moreover, a spindle of intermediate length has never been observed, suggesting “bistability” of the spindle^30^,^31^. Some of the discrepancies may be due to system-specific variations^4^. In the study on cultured human cells, spindles were monitored live with mCherry-tubulin without chromosome alignment as a marker^31^; thus, we suspect it may be difficult to score the near-metaphase spindles in this experiment. To assess whether kinesin-5 plays a role in metaphase spindle scaling in human cancer cells with temporal precision and robust statistics, we turned to immunofluorescence. CL1-5 cells were synchronized by double thymidine block, released into low dose of kinesin-5 inhibitor *S*-trityl-l-cysteine (STLC; the IC_50_ for STLC-induced mitotic arrest in HeLa cells is 0.7 μM)^32^ for 8 hr, and treated with MG132 for another 1.5 hr to shortly arrest the cells at metaphase. The cells were then fixed and stained for spindles and chromosomes, and only cells with well-aligned chromosomes were considered at metaphase. Weak inhibition of kinesin-5 activity dose-dependently decreased the metaphase spindle length in CL1-5 (Fig. 3B,C). In addition, with increasing concentration of STLC, there were less curved spindles in CL1-5 (Fig. 3D), and the aspect ratio approached that of CL1-0, reminiscent of the smaller, normal CL1-0 spindles (Fig. 3B,C). We observed a significant amount of monopolar spindles in CL1-5 cells treated with 0.7 μM STLC, consistent with the previously observed kinesin-5 threshold for monopolar-to-bipolar spindle transition^30^,^32^. Weak inhibition of kinesin-5 induced a similar reduction in the aspect ratio in MDA-MB-231 (Figure S8), suggesting that the variation in steady-state metaphase length is not specific to CL1-5. Additionally, we tested whether overexpression of kinesin-5 could lengthen CL1-0 spindles. To exclude the possibility that the fluorescent tag may perturb protein function, untagged kinesin-5 was overexpressed in CL1-0 using a bidirectional CMV vector (Fig. 3E). Ectopic kinesin-5 expression in CL1-0 was confirmed with qRT-PCR (Fig. 3E), and led to CL1-5-like metaphase spindles (Fig. 3F). Collectively, these data demonstrate that in human cancer cells, a physiologically relevant upregulation of the sliding motor, kinesin-5, can lead to lengthened, sometimes even curved, steady-state metaphase spindles. The less stringent constraints on genome integrity in cancers (particularly in the CL series) presumably allows for much more visibly deformed spindles as compared to other systems.

### Inhibition of kinesin-5 activity suppresses the speed of spindle elongation and post-mitotic migration in CL1-5

**Figure 4:**
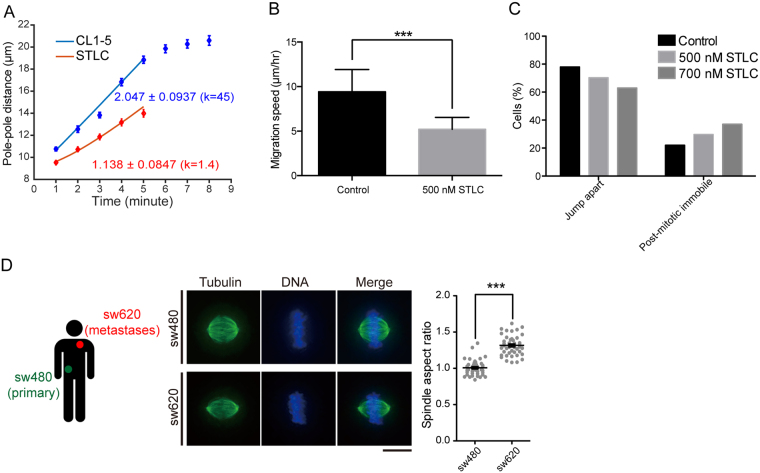
Perturbation of Spindle Dynamics and Its Implications in Cancer. (**A**) Experimental and theoretical plots of pole-pole distance as functions of time during anaphase B in CL1-5 with or without STLC perturbation. Blue: control; red: 0.5 μM STLC. Solid lines: theoretical curves (k = 45 and 1.4 for control and STLC, respectively). Experimental data are represented as the mean ± SEM (n = 10 and 15 for control and STLC, respectively). (**B**) Comparison of post-mitotic cell migration of CL1-5 with or without STLC perturbation. Total migration distances travelled in the post-mitotic four hr were analyzed in individual CL1-5 cells with indicated treatments. Data are represented as the mean ± SEM. n = 16 for each group. (**C**) STLC perturbation increases the percentage of post-mitotic, immobile CL1-5 cells. Data are represented as the mean ± SEM (n = 145, 350, and 108 for control, 0.5 μM, and 0.7 μM STLC, respectively). (**D**) (Left) representative immunofluorescence images of SW480 and SW620 spindles. Tubulin: green; DNA: blue. Scale bar: 10 μm. (Right) spindle aspect ratio of SW480 and SW620. n = 44 for each cell line.

Careful *in vitro* study has demonstrated that ensembles of kinesin-5 can exert pushing as well as braking forces depending on the orientation (i.e., parallel or anti-parallel) and relative motion of the cross-linked microtubules^33^. Specifically, in axons, where the overlapping microtubules are of the same polarity orientation (i.e., parallel), kinesin-5 acts as a brake to impose restrictions on axon growth^34^. In the mitotic spindle, where kinesin-5 is known to act between interpolar microtubules that are anti-parallel, it mainly provides pushing forces to assist in spindle pole separation^25^,^26^,^27^. However, it can also resist pole separation when the outward pulling forces are too strong: in worms, in which very large astral microtubule arrays and unusually strong Gα-dependent cortical pulling occur, kinesin-5 is evolved to serve as a rate-limiting brake for interpolar microtubule sliding to ensure that the anaphase forces are precisely balanced for proper DNA segregation^35^. Since somatic spindles in human cells contain only moderate astral microtubules (note that there still is dynein-dynactin-dependent cortical pulling in human somatic cells)^36^,^37^, we reasoned that the fast spindle elongation in CL1-5 may arise mainly from a higher kinesin-5-dependent pushing force that slides interpolar microtubules apart. Indeed, weak inhibition of kinesin-5 activity in GFP-tubulin-expressing CL1-5 largely reduced the spindle-elongation speed (Fig. 4A, linear regression slopes are 2.047 ± 0.0937 and 1.138 ± 0.0847 for normal and STLC-treated CL1-5 cells, respectively). To infer the interpolar kinesin-5-dependent pushing force, we employed a previously established theoretical model that describes pole-pole spacing as a balance of antagonistic forces exerted on the poles by sliding motor proteins and microtubule-depolymerizing enzymes^38^. The dependence of the elongation speed on the activity of kinesin-5 can be recapitulated by the force-balance model (Fig. 4A and Videos S6 and S7, see Experimental Procedures for details), suggesting that upregulation of kinesin-5 exerts a higher pushing force to speed up spindle elongation in the metastatic CL1-5. Next, we sought whether there is a correlation between kinesin-5 activity and post-mitotic cell migration. Indeed, weak inhibition of kinesin-5 also suppressed post-mitotic cell migration in CL1-5 (Fig. 4B; cell migration before entry into mitosis was not perturbed at this concentration). Some of the CL1-5 cells that were highly mobile before mitotic entry became transiently immobile after weak STLC-induced perturbation of mitosis (Fig. 4C). Together, these data suggested that upregulation of kinesin-5 provides a higher pushing force that contributes to a fast anaphase B, which correlates with a transiently elevated migration speed in CL1-5. This dynamic role may allow the upregulation of kinesin-5 and lengthened metaphase spindle to be selected in this metastatic clone.

### Correlation between spindle length and metastatic potential in colon cancer lines evolved and selected *in vivo*

Our data suggested that spindle architecture, or aspect ratio, may be able to serve as an *in vivo* biomarker for metastatic cancer clones. To assess whether a correlation exists between high aspect ratio and strong metastatic potential, we used SW480 and SW620 paired colon cancer cell lines derived from primary (*in situ*) and secondary (metastatic) tumors resected from a single patient as a model for *in vivo* cancer evolution and selection^39^. The aspect ratio of SW620 was significantly higher than that of SW480 (Fig. 4D), supporting our idea of employing aspect ratio as a novel biomarker for metastatic cancers. A slight upregulation of kinesin-5 expression in SW620 was found with qRT-PCR (Figure S9), though there is a possibility that other sliding motors may also contribute to the alteration of the aspect ratio^19^.

## Discussion

During natural evolution, the bipolar spindle is believed to scale with cell size to execute efficient and accurate search-and-capture of the kinetochores to divide the genome^1^,^2^,^3^,^4^,^5^. In this study, we focused on this aspect in the context of cancer evolution toward metastasis, using two subclones, CL1-0 and CL1-5, from our previously established model cell lines^12^. We found that while average spindle length correlated very well with the average cell size for randomly selected reference cell lines (A549, MDAMB-231, ARPE-19 and CL1-0, the least metastatic clone within the CL series), the highly metastatic CL1-5 evolved a rather lengthened steady-state metaphase spindle given its relatively smaller cell size. Microarray analysis and qRT-PCR confirmed a physiologically relevant upregulation of kinesin-5 in CL1-5, and dose-dependent pharmacological inhibition of kinesin-5 activity shortened the CL1-5 spindles. Conversely, ectopic expression of kinesin-5 led to lengthened spindles in CL1-0. The reduced demand on faithful DNA segregation during cell division in the CL series presumably allows for lengthened—and sometimes even curved—spindles to exist at metaphase when kinesin-5 levels are elevated.

What might be the possible reasons for the upregulation of kinesin-5 and lengthened steady-state metaphase spindle to be selected in CL1-5? Since kinesin-5 is a motor protein that cross-links interpolar microtubules and slides them apart^25^,^26^, we looked at spindle dynamics and found that CL1-5 accommodated a faster spindle elongation during anaphase B, which, according to the force-balance model, corresponds to a stronger kinesin-5-dependent pushing force. This correlates with a transiently elevated speed and directional persistence of post-mitotic cell migration, pointing to a working model that the motor-driven forces that push two spindle poles apart during anaphase B might transiently promote migration, thus conferring an advantage in metastasis. We tested an extreme case of this idea by inducing monopolar-spindle CL1-5 cells, i.e., no outward force, using a saturating concentration of STLC (Figure S10A). We then forced cells to exit mitotic arrest with VX-680, an Aurora-B inhibitor, and found that the post-mitotic peak in percentage of migration distance was lost (Figure S10B), supporting the model. Further experiments, for instance using micropillar arrays^40^ to experimentally measure how spindle-mediated force is translated onto the daughter cells, would be critical to strengthen the model. Since kinesin-5 can also recruit and sort microtubules into bundles of parallel microtubules anchored at the spindle poles^27^, a tempting alternative would be that upregulation of kinesin-5 may lead to stronger/larger astral microtubule arrays, such that plus ends of the astral microtubules can be in better contact with or position actin-related proteins at the cortex, e.g., via EB1/APC/mDia/Rho^41^ or CLIP-170/IQGAP1/Cdc42/Rac1^42^, to promote actin-mediated cell migration immediately after cell division. Those models are not mutually exclusive, and their contribution to this process can be further clarified in the future.

As many kinesin-5 inhibitors have failed to show profound efficacy as a cancer monotherapy in clinical trials^43^, our data suggest that these inhibitors may still possess alternative therapeutic benefits, for instance, by preventing the devastating metastasis. Another clinical implication of our data is that the spindle aspect ratio can serve as a cellular biomarker for metastatic cancer clones. Indeed, overexpression of HSET, a human kinesin-14 that also acts to slide microtubules apart, led to lengthened metaphase spindles in human cancer cells^19^, suggesting that alteration of the aspect ratio via microtubule crosslinkers is not specific to kinesin-5. That is, there may be other microtubule sliders that are upregulated to lengthen spindles in the progression of cancer toward metastasis. The similar correlation between high aspect ratio and strong metastatic potential in SW480 and SW620 suggests that the phenomenon is also not specific to the CL series or one type of cancer. Tissue sections and the ongoing development of *in vivo* probes and imaging methods will allow this idea to be tested and of true prognostic value in the future.

## Experimental Procedures

### Cell Lines, Drugs, and Synchronization

CL1-0, CL1-1, CL1-2, CL1-5, and A549 cells were cultured in RPMI 1640 with 10% FBS (HyClone) and 1% penicillin/streptomycin (Gibco) at 3 °C in a 5% CO_2_ water-jacketed incubator (Thermo Scientific). ARPE-19 cells were cultured in DMEM/F12 with 10% FBS and 1% penicillin/streptomycin at 37 °C in a 5% CO_2_ water-jacketed incubator. MDA-MB-231, SW480 and SW620 cells were cultured in Leibovitz’s L-15 supplemented with 10% FBS and 1% penicillin/streptomycin at 37 °C without CO_2_ following ATCC recommendations. Stable GFP-tubulin-expressing CL1-0 and CL1-5 were generated using LentiBrite GFP-Tubulin Lentiviral Biosensor (Merck Millipore). *S*-Trityl-l-cysteine and MG132 (Merck Millipore) and VX680 (LC Laboratory) were used at specified concentrations. For synchronization, cells were incubated in medium containing 2 mM thymidine (Sigma) for 19 hr, in regular medium for 9 hr, and in medium containing 2 mM thymidine for 16 hr. The cells were then released into regular medium for 7 hr (for collection at G2 phase) or into specified drug treatments.

### Immunofluorescence

Cells were fixed with 3.7% formaldehyde at 37 °C for 20 min, permeabilized with 0.3% Triton X-100 in PBS at room temperature for 10 min, blocked in 1% BSA/PBS at room temperature for 30 min, and incubated in 1% BSA/PBS with FITC-conjugated anti-α-tubulin (Sigma) at 4 °C for 2 hr. Slides were washed thrice in 0.1% Triton X-100/PBS and mounted using ProLong Gold Antifade Mountant with DAPI (Life Technologies). Images were acquired using a DeltaVision Core microscope (100× oil/NA1.40) (Applied Precision). Statistical analysis using Student’s *t-*test was performed using GraphPad Prism.

### Time-lapse Imaging

For tracking of single-cell migration, images were recorded every 20 or 30 min using an inverted fluorescence microscope (Axio Observer Z1, 20×/NA0.4; Carl Zeiss) in a humidified chamber maintained at 37 °C with 5% CO_2_. For tracking of spindle pole separation, images were recorded every minute using a DeltaVision Core microscope (10× oil/NA1.40; Applied Precision). DNA was stained with 1 μg/ml Hoechst 33342 (Life Technologies). Migration distance, angles, and pole-to-pole distance were measured manually using ImageJ.

### qRT-PCR

Total RNA was isolated with an RNeasy Mini Kit (QIAGEN) and reverse-transcribed with SuperScript III reverse transcriptase (Invitrogen). qRT-PCR reactions were carried out with the SYBR Green qPCR kit (KAPA Biosystems) on a CFX96 qPCR machine (Bio-Rad). Relative mRNA levels were calculated according to the ΔΔCt method; *GAPDH* was used as reference gene. The following primers were used: KIF11_F: CCAGCAAGCTGCTTAACACAGT; KIF11_R: CTTCTAGCATGGCCTTTTGCTT; GAPDH_F: TGGTGAAGCAGGCGTCGGAG; GAPDH_R: GGTGGGGGACTGAGTGTGGC.

### Cloning and Transfection

KIF11 human cDNA (Origene, SC110939) was cloned into pBI-CMV1 (Clontech), together with mCherry to facilitate scoring of transfected cells. CL1-0 cells were transfected using Lipofectamine 3000 (Invitrogen), harvested after 48 hr, and examined by immunofluorescence microscopy.

### Transwell Migration Assay

CL1-0 or CL1-5 cells incubated with 5% FBS-/RPMI were seeded into 8.0-μm pore-size Transwell migration chambers (Corning), and 10% FBS/RPMI was added to the lower chambers. The cells were left to migrate for 24 hr, fixed in 3.7% formaldehyde, and stained with DAPI (1 μg/ml) in PBS. For every Transwell, four images were taken from random fields using an Axio Observer Z1 (10×/NA0.4; Carl Zeiss, Germany), and cells were counted manually.

### Mathematical Modeling

Computer programs were written with MATLAB (MathWorks). Anaphase-B dynamics was described with a previously established theoretical model that describes pole-pole spacing (*S*(*t*)) as a balance of antagonistic forces exerted on the poles by sliding motor proteins and microtubule-depolymerizing enzymes^38^. Essentially, this force-balance model consists of coupled differential equations based on the following set of three core equations that describes the central spindle as antiparallel overlapping microtubules:


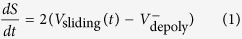



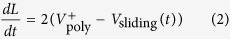



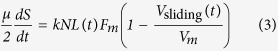


where *k* denotes the number of motors per interpolar microtubule-overlap length, which is the parameter that was varied to determine changes in kinesin-5 activity. Because the model only describes anaphase-B dynamics with a fixed steady-state metaphase length^38^, we introduced a *k*-dependent offset (*offset* = 0.3327*log_10_(0.1772*k) in the initial spindle length to accommodate our experimental observations.

## Additional Information

**How to cite this article**: Yang, C.-F. *et al.* Kinesin-5 Contributes to Spindle-length Scaling in the Evolution of Cancer toward Metastasis. *Sci. Rep.* **6**, 35767; doi: 10.1038/srep35767 (2016).

## Supplementary Material

Supplementary Information

Supplementary table

Supplementary video 1

Supplementary video 2

Supplementary video 3

Supplementary video 4

Supplementary video 5

Supplementary video 6

Supplementary video 7
