# Supplementary material for: Kinesin-5 Contributes to Spindle-length Scaling in the Evolution of Cancer toward Metastasis
Source: Sci Rep. 2016 Oct 21;6:35767. doi: 10.1038/srep35767 (PMC5073351; doi:10.1038/srep35767)
Supplement: Supplementary Information [file srep35767-s1.pdf]

Supplementary Information

Kinesin-5 Contributes to Spindle-length Scaling in the Evolution of Cancer toward Metastasis

Ching-Feng Yang*, Wan-Yu Tsai*, Wei-An Chen*, Kai-Wen Liang, Cheng-Ju Pan, Pei-Lun Lai, Pan-Chyr Yang, Hsiao-Chun Huang.

*These authors contributed equally to this work.

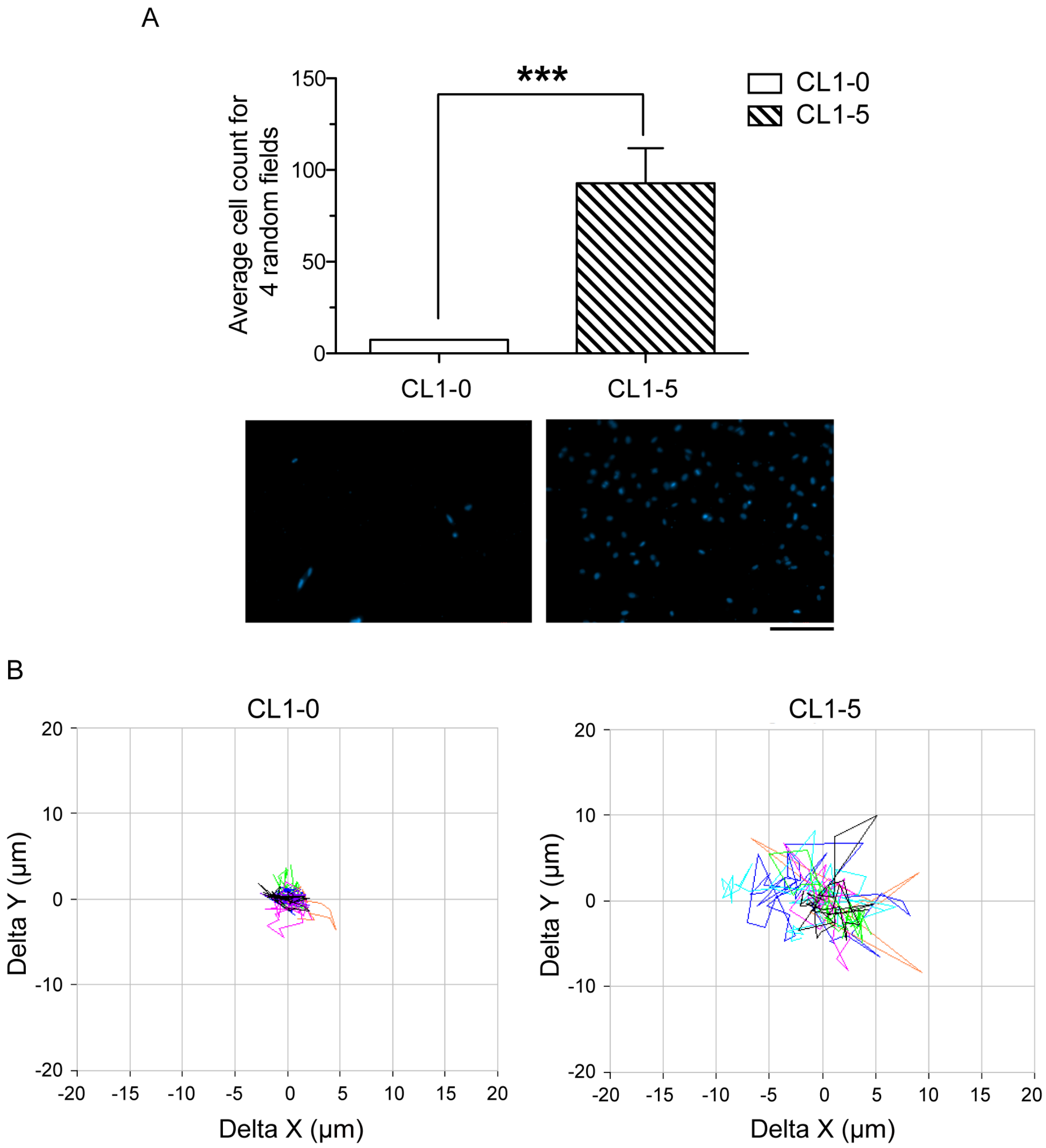

Figure S1. ***In vitro*** **Migration Activity of CL1-0 and CL1-5.**

(A) Migration activity was measured by Transwell assay. Cell counts were averaged from four randomly selected fields. Bar graph represents average ± SEM from two independent experiments ($P < 0.001$, Student's t test). Representative field images are shown at bottom. (B) Migration of individual cells was monitored using time-lapse microscopy. Data were presented as overlays of representative trajectories.

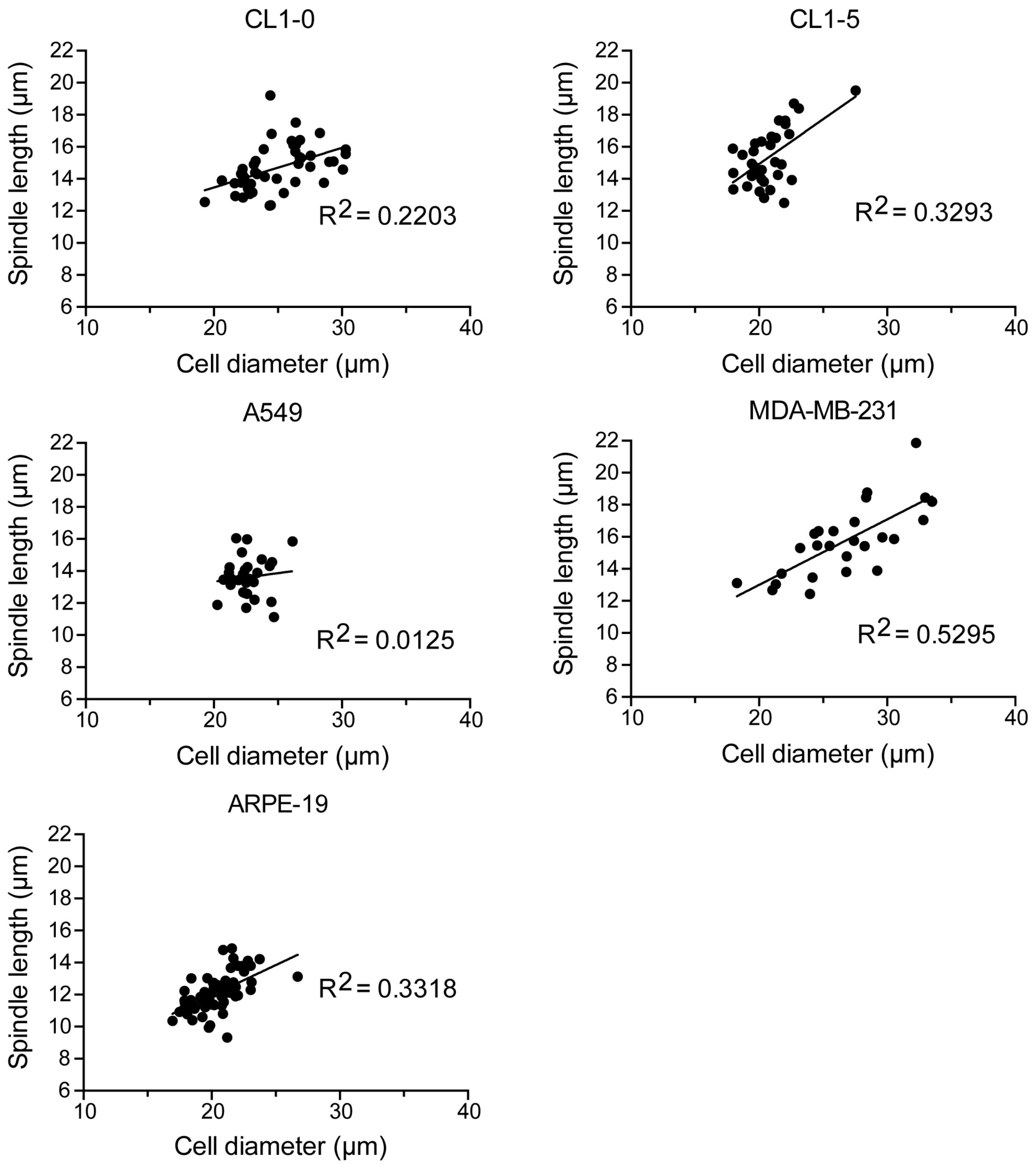

Figure S2. Metaphase Spindle Length Plotted Against Cell Diameter for Five Human Cancer Cell Lines (CL1-0, CL1-5, A549, MDA-MB-231 and ARPE-19).

Each dot represents a single cell. Linear regression lines and R^2 values are shown.

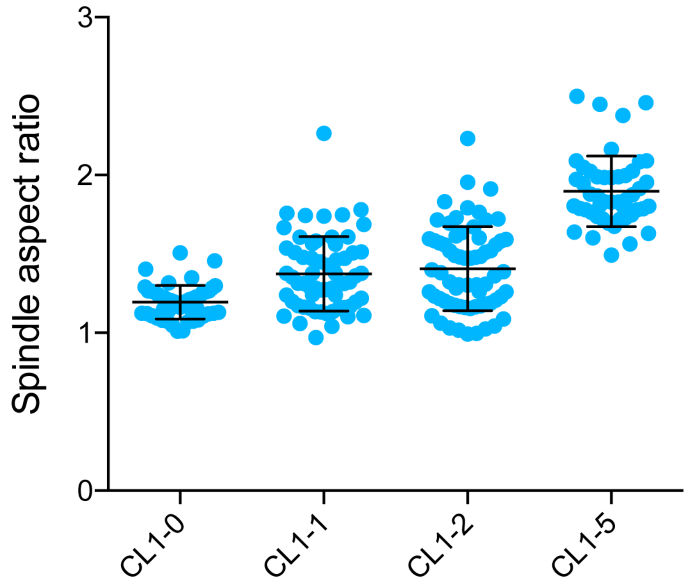

Figure S3. Spindle Aspect Ratio of CL1-0, CL1-1, CL1-2 and CL1-5.

Average aspect ratio is 1.32 ± 0.14 (n = 48), 1.37 ± 0.23 (n = 62), 1.41 ± 0.26 (n = 67) and 1.89 ± 0.22 (n = 48) for CL1-0, CL1-1, CL1-2 and CL1-5, respectively.

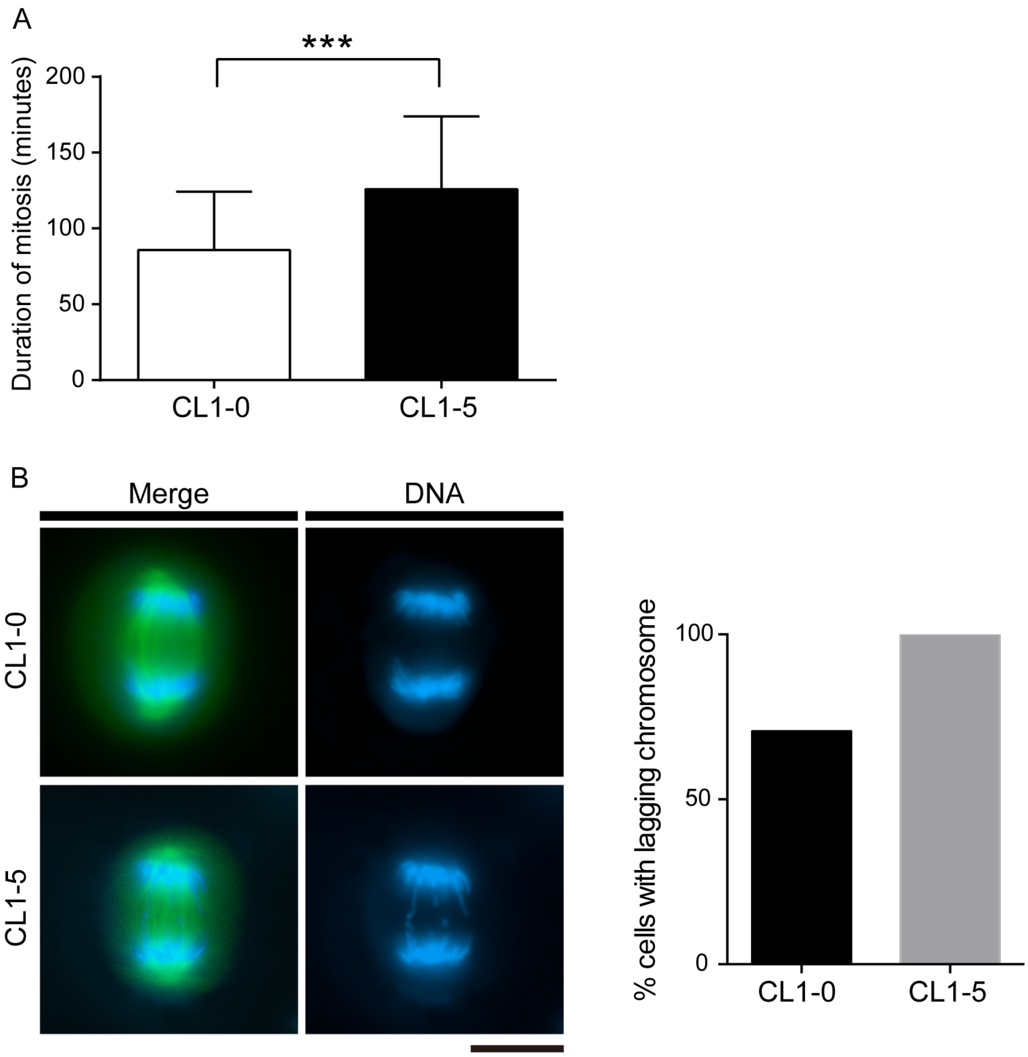

Figure S4. Duration of Mitosis and Chromosome Segregation in CL1-0 and CL1-5.

(A) Duration of mitosis measured from cells rounding up until flattening out again. Average duration is 85.7 minutes (n=77) and 125.9 minutes (n=108) for CL1-0 and CL1-5, respectively. (B) Analysis of chromosome segregation. Lagging chromatids were scored during anaphase by time-lapse imaging. Representative images are shown at left. Percentage of lagging chromatids is 70.6% (n=17) and 100% (n=12) for CL1-0 and CL1-5, respectively. Scale bar: 20 μm.

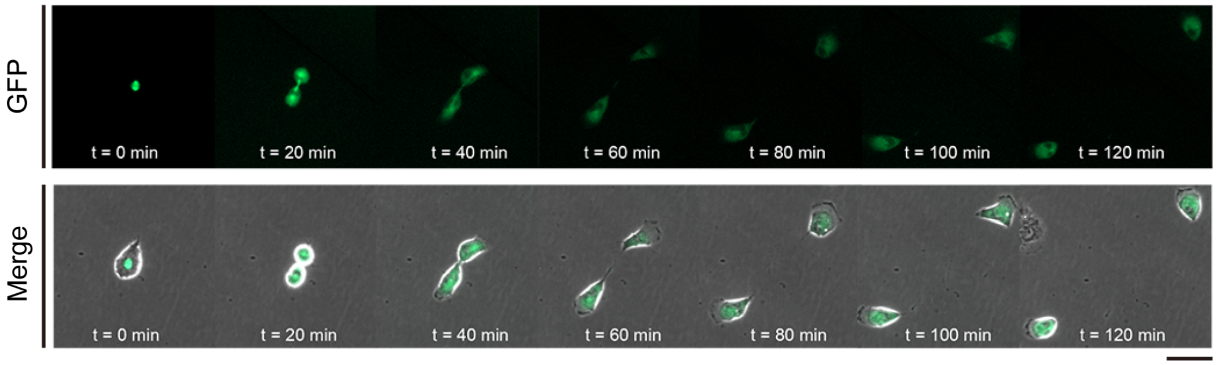

Figure S5. Representative Time-lapse Images of Tilted-CL1-5 Migration Following Cytokinesis.

Cleavage furrow (phase channel) and spindle/midbody (GFP channel) were used to judge the axis of cell division in GFP-tubulin-expressing CL1-5 cells. Images were taken every 30 min. Scale bar: 50 μm.

	Total cells analyzed	Number of typical CL1-5 (mobile) cells	Number of CL1-0-like (immobile) cells	Percentage of CL1-0-like cells
CL1-5 passage #1	94	66	28	29.79
CL1-5 passage #2	115	74	41	35.66
CL1-5 passage #3	51	38	13	25.49
			Ave ± STDV	30.31 % ± 4.17 %

Figure S6. Quantification of CL1-5 heterogeneity.

The percentage of CL1-0-like (immobile) cells in three independent CL1-5 passages were scored using time-lapse microscopy.

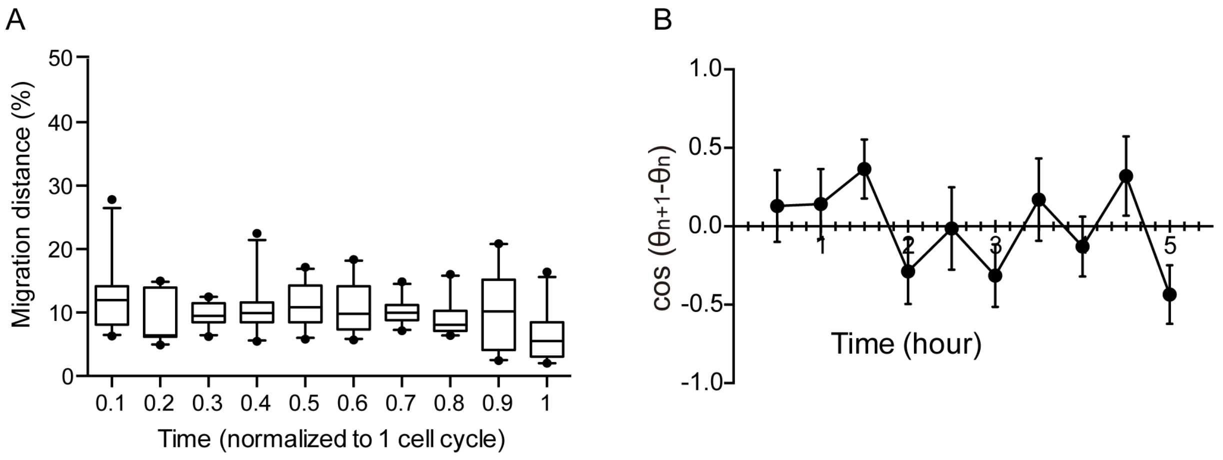

Figure S7. Analysis of CL1-0 migration.

(A) Boxplot of the speed of CL1-0 single-cell migration profiled over the course of cell cycle (n = 10 cells). (B) Transient directional persistence of CL1-0 migration following mitosis (n = 10 cells).

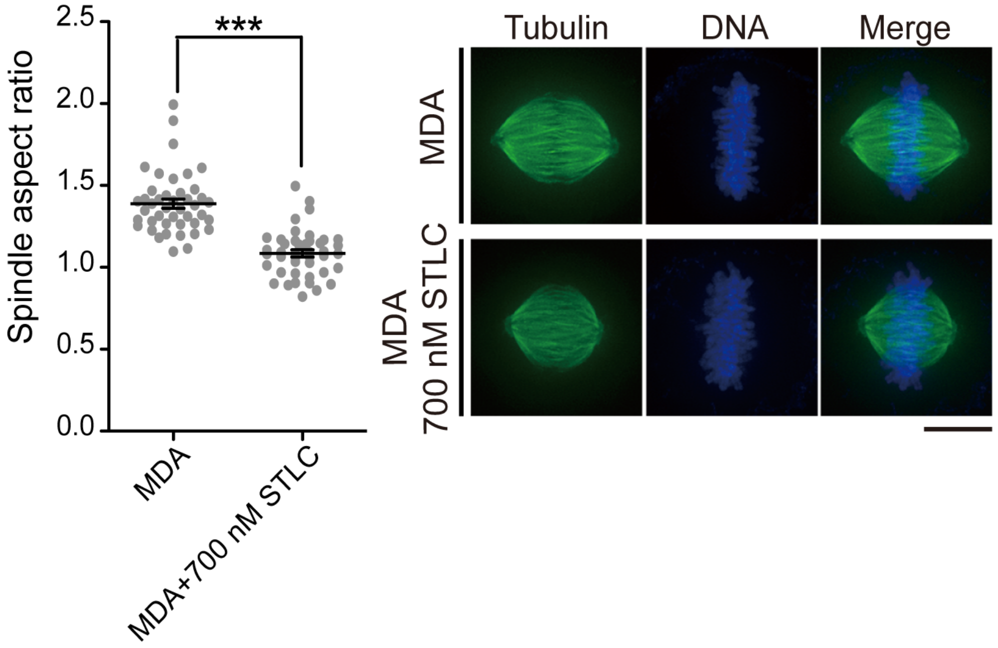

Figure S8. Spindle Aspect Ratio and Representative Immunofluorescence Images of MDA-MB-231 with or without 0.7 μM STLC.

Average aspect ratio is 1.38 ± 0.18 (n = 43) and 1.08 ± 0.74 (n = 43) for control and 0.7 μM STLC-treated cells, respectively. $P < 0.001$; Student's t test. Tubulin: green; DNA: blue. Scale bar: 10 μm.

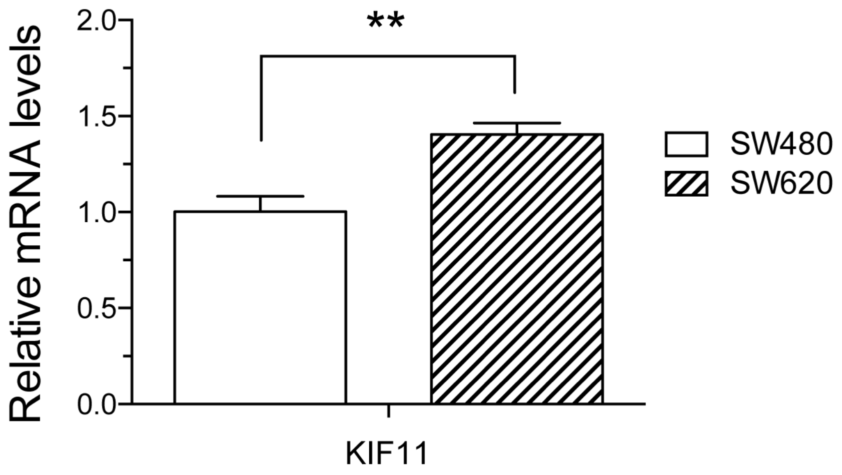

Figure S9. Comparison of Kinesin-5 expression in SW480 and SW620.

Level of kinesin-5 mRNA analyzed by qRT-PCR ($n = 3$ independently experiments, $P < 0.001$; Student's t test).

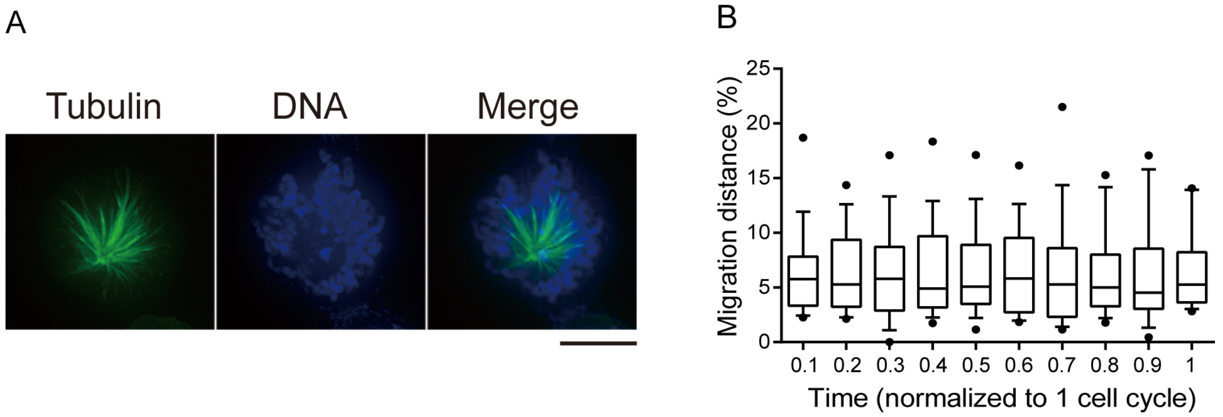

Figure S10. Migration of CL1-5 forced to exit from monopolar-spindle mitotic arrest.

(A) Representative immunofluorescence images of CL1-5 monopolar spindles. CL1-5 were treated with 5 μM STLC and then fixed and stained for tubulin (green) and DNA (blue). Scale bar: 10 μm. (B) Boxplot of the speed of slipped-CL1-5 migration profiled over the course of cell cycle (n=16 cells).

Videos S1–3. 3D reconstructed immunofluorescence images showing CL1-0 (Video S1), CL1-5 straight (Video S2), and CL1-5 curved (Video S3) spindles.

Videos S4-5. Representative CL1-0 (Video S4) and CL1-5 (Video S5) cells at prometaphase.

Videos S6–7. Computer simulation of elongating anaphase B spindles with k = 1.4 (Video S6) and k = 45 (Video S7).

Table S1. Differentially Expressed Genes in G2-phase CL1-5.
